# In silico recognition of a prognostic signature in basal-like breast cancer patients

**DOI:** 10.1371/journal.pone.0264024

**Published:** 2022-02-15

**Authors:** Federica Conte, Pasquale Sibilio, Anna Maria Grimaldi, Marco Salvatore, Paola Paci, Mariarosaria Incoronato

**Affiliations:** 1 Institute for Systems Analysis and Computer Science “Antonio Ruberti”, National Research Council, Rome, Italy; 2 Department of Translational and Precision Medicine, Sapienza University of Rome, Rome, Italy; 3 IRCCS Synlab SDN S.p.A., Naples, Italy; 4 Department of Computer, Control and Management Engineering, Sapienza University of Rome, Rome, Italy; Sapporo Ika Daigaku, JAPAN

## Abstract

**Background:**

Triple-negative breast cancers (TNBCs) display poor prognosis, have a high risk of tumour recurrence, and exhibit high resistance to drug treatments. Based on their gene expression profiles, the majority of TNBCs are classified as basal-like breast cancers. Currently, there are not available widely-accepted prognostic markers to predict outcomes in basal-like subtype, so the selection of new prognostic indicators for this BC phenotype represents an unmet clinical challenge.

**Results:**

Here, we attempted to address this challenging issue by exploiting a bioinformatics pipeline able to integrate transcriptomic, genomic, epigenomic, and clinical data freely accessible from public repositories. This pipeline starts from the application of the well-established network-based SWIM methodology on the transcriptomic data to unveil important (switch) genes in relation with a complex disease of interest. Then, survival and linear regression analyses are performed to associate the gene expression profiles of the switch genes with both the patients’ clinical outcome and the disease aggressiveness. This allows us to identify a prognostic gene signature that in turn is fed to the last step of the pipeline consisting of an analysis at DNA level, to investigate whether variations in the expression of identified prognostic switch genes could be related to genetic (copy number variations) or epigenetic (DNA methylation differences) alterations in their gene loci, or to the activities of transcription factors binding to their promoter regions. Finally, changes in the protein expression levels corresponding to the so far identified prognostic switch genes are evaluated by immunohistochemical staining results taking advantage of the Human Protein Atlas.

**Conclusion:**

The application of the proposed pipeline on the dataset of The Cancer Genome Atlas (TCGA)-Breast Invasive Carcinoma (BRCA) patients affected by basal-like subtype led to an *in silico* recognition of a basal-like specific gene signature composed of 11 potential prognostic biomarkers to be further investigated.

## Introduction

Breast cancer (BC) is the most common female cancer and despite important advances in early detection and research development, it continues to be the second leading cause of death in women worldwide [1]. Triple-negative BC (TNBC) accounts for a minority of all diagnosed BCs (15–20%) [2]. It is a subtype with a heterogeneous nature, defined by the low or absent expression of estrogen (ER), progesterone (PR) receptors and the lack of expression of the human epidermal growth factor (EGF) receptor-2 (HER2) receptors [3]. These cancers differ from other BC subtypes in that they grow and spread faster, have limited treatment options (typically treated with chemotherapy) and their metastatic pattern spread with a higher likelihood of brain and lung involvement and less frequently with bone lesions. Relapse is common in TNBC, usually in the first 5 years, leading to the poorest survival outcomes between all BC subtypes [4]. Currently, there are not available widely-accepted prognostic markers to predict outcomes in TNBC patients. TNBC is often used as a surrogate for identifying the aggressive basal-like BC subtype. Although the two patterns share many similarities, biologically they are not the same, but both are associated with poor clinical outcomes. Therefore, the development of new prognostic indicators for basal-like subtype represents an unmet clinical challenge that might be of benefit to the clinical management of this disease. To achieve this goal, we started from data extracted from our recent computational analysis of BC phenotypes [2]. In that study, we exploited The Cancer Genome Atlas (TCGA)-Breast Invasive Carcinoma (BRCA) dataset applying a network-based tool named SWItch Miner (SWIM), which predicts important (switch) genes within the co-expression network that regulate disease state transitions. The transcriptomic profile of BC patients was stratified into BC subtypes according to the well-established Immunohistochemistry (IHC) (Luminal A, Luminal B, Her2 positive and Triple-negative) and genetic (PAM50; Luminal A, Luminal B, Her2 positive and Basal-like) classification, to identify switch genes shared among four subtypes and those specific for each subtype. We focused our attention on shared switch genes to identify a common BC disease module univocally altered in all BC subtypes, leaving for a next deepening the understanding of the clinical utility of switch genes deregulated in a subtype-specific manner. So, here we focused our attention on switch genes specific for the most aggressive subtype (basal-like) to screen which of them, deregulated in BC patients, significantly associated with the survival of basal-like affected subjects. Correlation analyses were performed, and the results were complemented with further studies at both DNA and protein level, to investigate whether variations in the expression of identified prognostic switch genes could be related to genetic (copy number variations), epigenetic (DNA methylation differences), and transcription factor activities. Also, changes in the protein expression levels were evaluated by immunohistochemical staining results taking advantage of the Human Protein Atlas. Overall, our findings led to an *in silico* recognition of a basal-like prognostic gene signature composed of 11 genes to be further investigated.

## Results

### Study design

**Fig 1:**
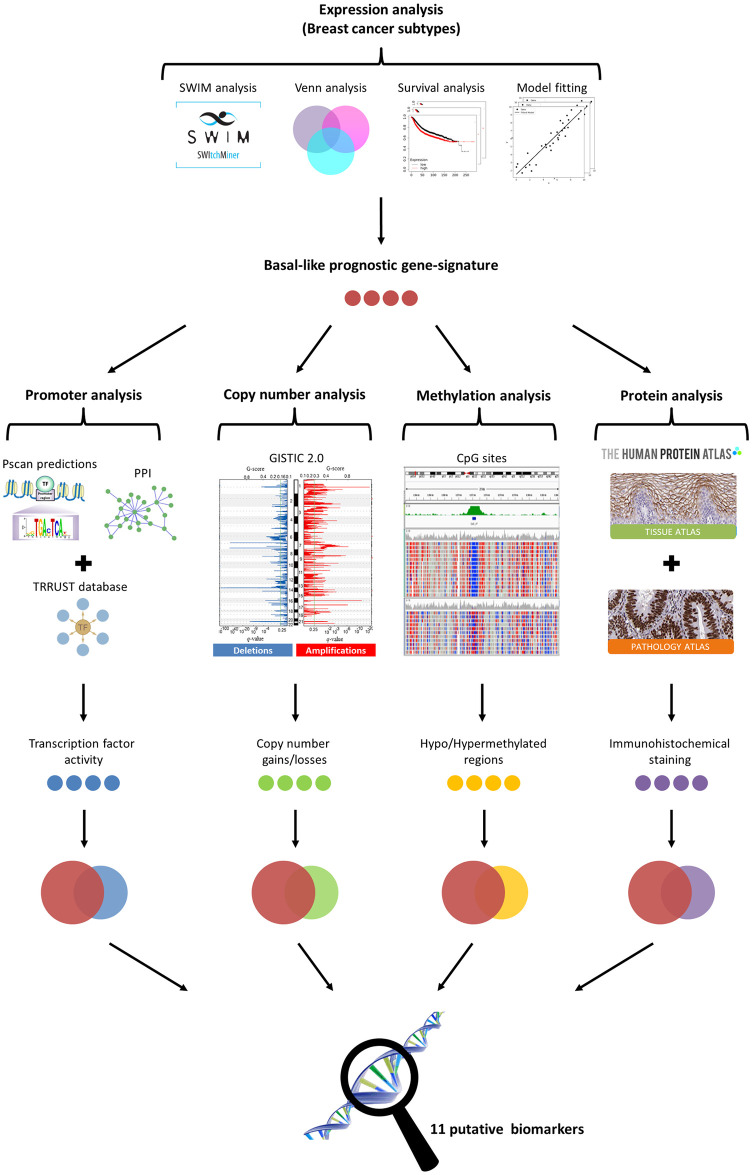
Study design. The figure depicts the schematic of the methodology applied in this study.

In our recent paper [2], we analysed a total of 505 BC subjects (229 Luminal A, 120 Luminal B, 58 HER2-enriched, and 98 Basal-like) and we identified a total of 108 switch genes (S1 Table) that were specific for the most aggressive BC subtype, i.e. the basal-like subtype [5–7]. In the present study, we aim to predict important prognostic biomarkers among these basal-like specific switch genes. A schematic for our study design is depicted in Fig 1.

### Prognostic value of basal-like specific switch genes

**Fig 2:**
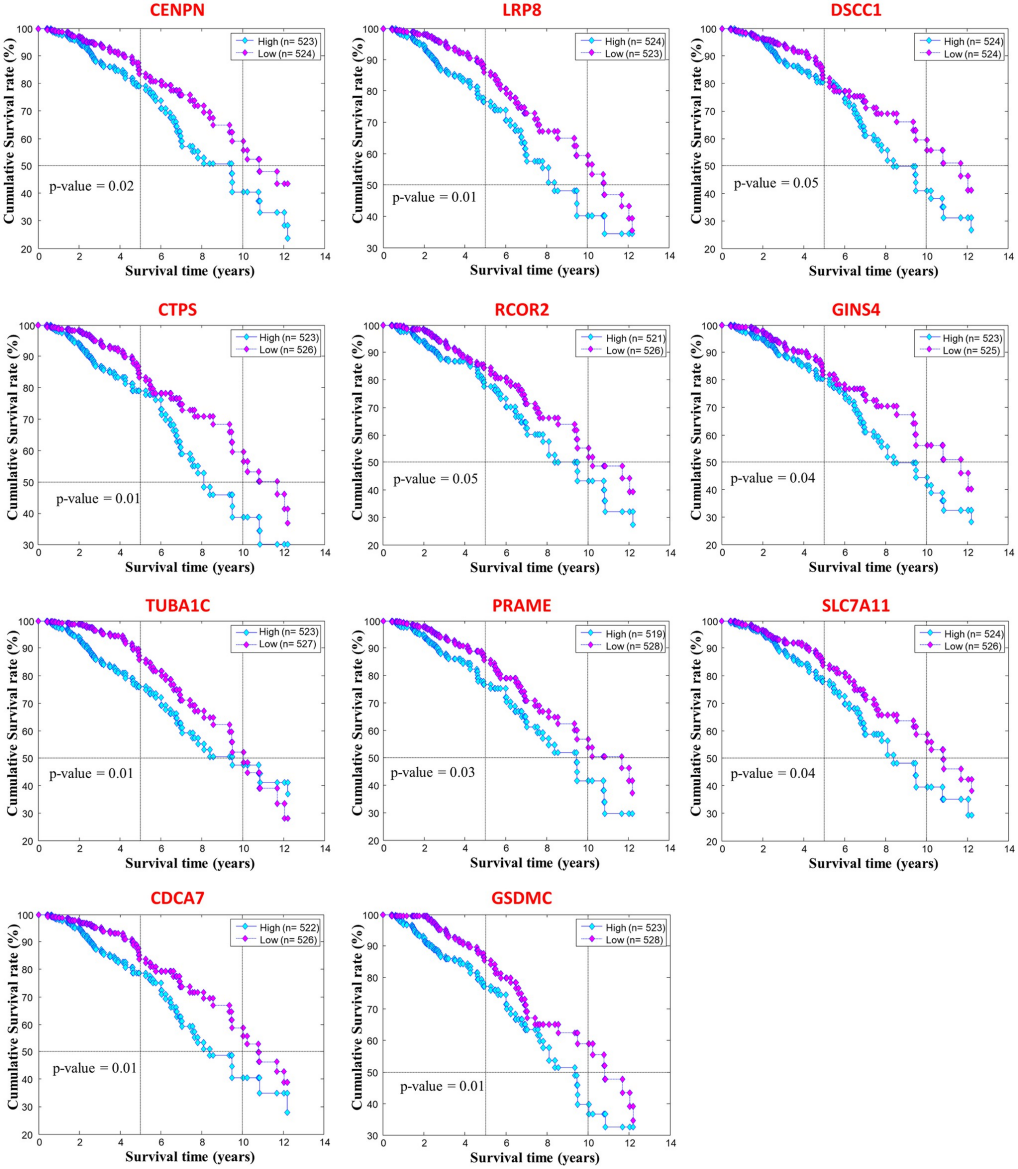
Switch genes with an unfavourable prognostic value from the survival analysis on TCGA data. Kaplan-Meier analyzes to evaluate the correlations between the expression of the basal-like specific switch genes and the OS in TCGA breast invasive carcinoma patients. Low- and high-expression groups refer to patients with expression levels lower and greater than the 50th percentile, respectively.

In order to study the clinical relevance of the basal-like specific switch genes with respect to the patients’ survival, we exploited their expression profiles to perform the Kaplan-Meier analysis. We used the RNA-sequencing data available on the TCGA to stratify BC patients in two groups according to the expression levels of the 108 basal-like specific switch genes. Thus, for each switch genes, low (high)-expression groups refer to patients with the expression level of that gene lower (greater) than the median of its expression values across all BC patients. Then, a log-rank test was performed to assess a statistical significance (p-value) to each gene: the lower the p-value, the better the separation between the two prognosis groups. Switch genes with log-rank p-values less than 0.05 were candidate as potential biomarkers for predicting the survival rate of breast cancer patients. We found a total of 15 basal-like specific switch genes that were significantly associated (p-values ≤ 0.05) with BC patients’ prognosis. Among them, 11 switch genes (i.e., CENPN, LRP8, DSCC1, CTPS, RCOR2, GINS4, TUBA1C, PRAME, SLC7A11, CDCA7, GSDMC) appeared to be an unfavourable prognostic gene (Fig 2), suggesting that their higher expression could be associated with poorer BC patients’ overall survival (OS). The other four switch genes (i.e., NXNL2, PHGR1, LOC389033, C10orf79) appeared as a favourable prognostic gene since their high expression correlated with a better clinical outcome (S1 Fig). Hereafter, we focused only on the 11 basal-like specific switch genes whose activation appeared to be associated with the worst prognosis. Their clinical relevance was also confirmed using other BC datasets collected in the Kaplan-Meier plotter website [8] (Table 1, RNA level).

**Table 1:** Summary of the properties of the basal-like prognostic biomarkers.

	DNA			RNA					Protein
	TFs	CNVs	Methylation	SWIM	KM analysis (log rank p-value)		model fitting (index R^2^)		IHC staining
	TRRUST/Pscan/PPI	TCGA	TCGA	TCGA	TCGA	other datasets	subtype	stage	HPA
CENPN	*NRF1*	amp in BL/del in LumA	hypo in BL	switch genes	0.02	4.9E-6	0.99	0.96	not available*
LRP8	*HIC1*	amp in BL/del in LumA	-	switch genes	0.01	2.4E-4	0.98	0.63*	more expressed in BC
DSCC1	*HMBOX1*	amp in BL	-	switch genes	0.05	3.5E-8	0.95	0.78	more expressed in BC
CTPS	*MYC*, *TWIST1*-*2*, *NRF1*	amp in BL/del in LumA	hypo in BL	switch genes	0.01	8.2E-5	0.94	0.72	more expressed in BC
RCOR2	-	-	-	switch genes	0.05	4.3E-3	0.93	0.47*	more expressed in BC
GINS4	-	-	-	switch genes	0.04	6.4E-3	0.90	0.68	more expressed in BC
TUBA1C	*TP53*, *NFKB1*	del in BL	-	switch genes	0.01	1.3E-6	0.89	0.76	more expressed in BC
PRAME	*NRF1*, *SOX9*, *RARA*	amp in BL/del in LumA	hypo in BL	switch genes	0.03	9.9E-6	0.83	0.76	not available*
SLC7A11	-	-	-	switch genes	0.04	0.03	0.80	0.46*	not available*
CDCA7	*MYC*, *E2F1*	amp in BL	-	switch genes	0.01	1.3E-4	0.73	0.32*	not available*
GSDMC	-	amp in BL	hypo in BL	switch genes	0.01	4.9E-4	0.64*	0.05*	not available*

Abbreviations: TFs, Transcription Factors; CNVs, Copy Number Variations; KM, Kaplan-Meier; IHC, Immunohistochemistry; PPI, protein-protein interactions; TCGA, The Cancer Genome Atlas; HPA, Human Protein Atlas; BC, Breast Cancer; BL, Basal-like; LumA, Luminal A; amp, amplified; del, deleted; hypo, hypomethylated. Asterisk (*) was used to highlight values not satisfying the chosen thresholds as well as not available data.

### Overexpression of the basal-like prognostic biomarkers

**Fig 3:**
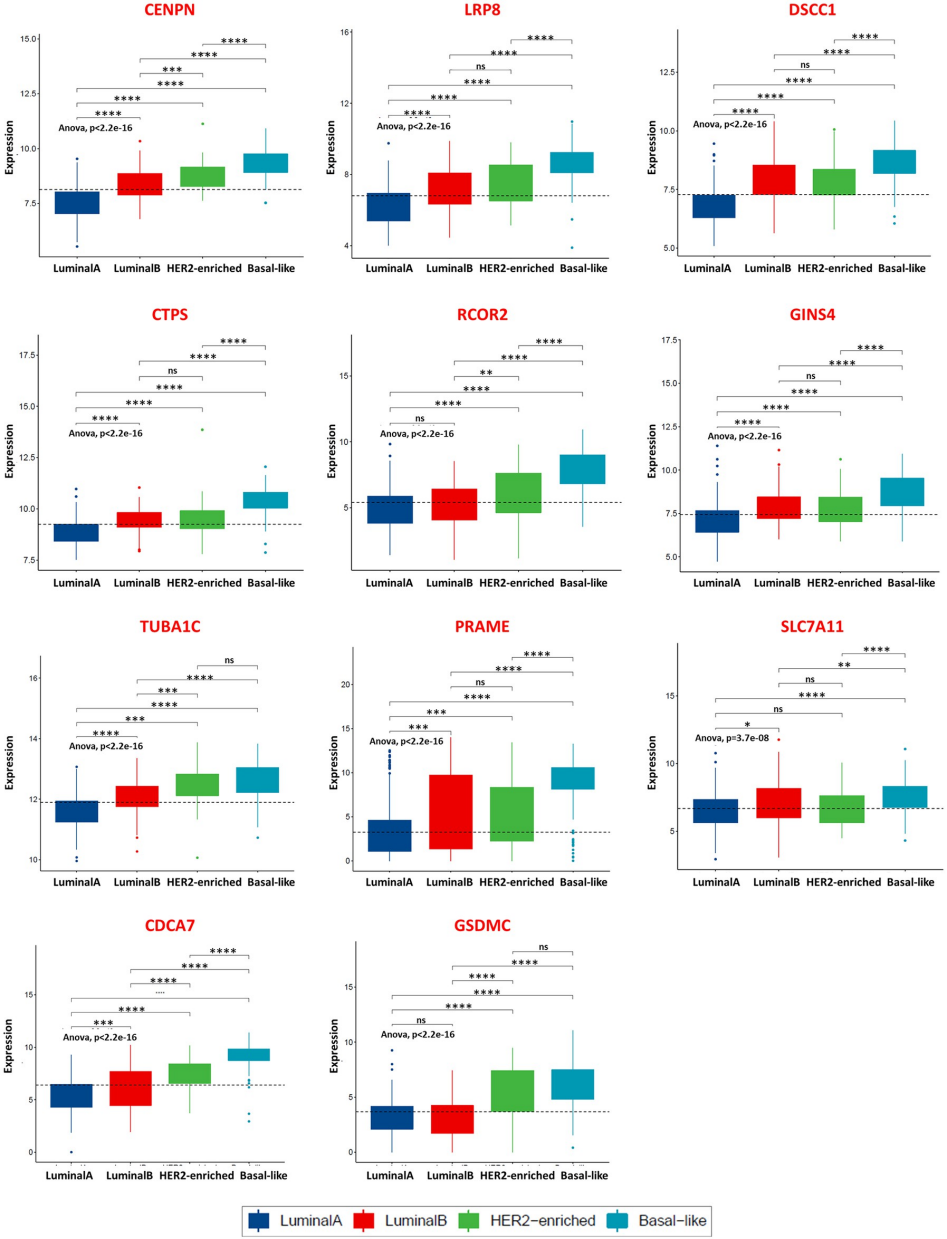
Expression of the switch genes with an unfavourable prognostic value across the PAM50 breast cancer subtypes. Gene expression levels of the 11 basal-like specific switch genes point out from the Kaplan-Meier survival analysis in TCGA breast invasive carcinoma patients affected by the four BC subtypes of PAM50 classification. The black dashed line reported in each plot indicates the median value used in the Kaplan-Meier survival analysis to split the low-expression and high-expression group. One-way ANOVA test was used to compare the means of the selected genes among the patients’ groups. T-test was used to perform multiple pairwise-comparisons and statistical significance was indicated by the star symbols (i.e., ns: p > 0.05, *: p ≤ 0.05, **: p ≤ 0.01, ***: p ≤ 0.001, ****: p ≤ 0.0001).

A differential expression analysis showed that the 11 basal-like specific switch genes, whose unfavourable prognostic value was statistically significant from the previous survival analysis, were all up-regulated in the basal-like cancer condition compared to the normal condition (S2 Fig). Yet, by performing an ANOVA test and multiple pairwise-comparisons among all the BC subtypes, we found that each comparison is statistically significant and the expression value of the 11 basal-like specific switch genes is greater in the basal-like versus the others BC subtypes and always greater than the median used in the KM survival analysis, leading to an association between worst prognosis patients (high-expression groups in the KM plots) and basal-like affected subjects (Fig 3). Taken all together, these findings prompted us to identify these 11 switch genes as potential prognostic biomarkers for basal-like subtype.

**Fig 4:**
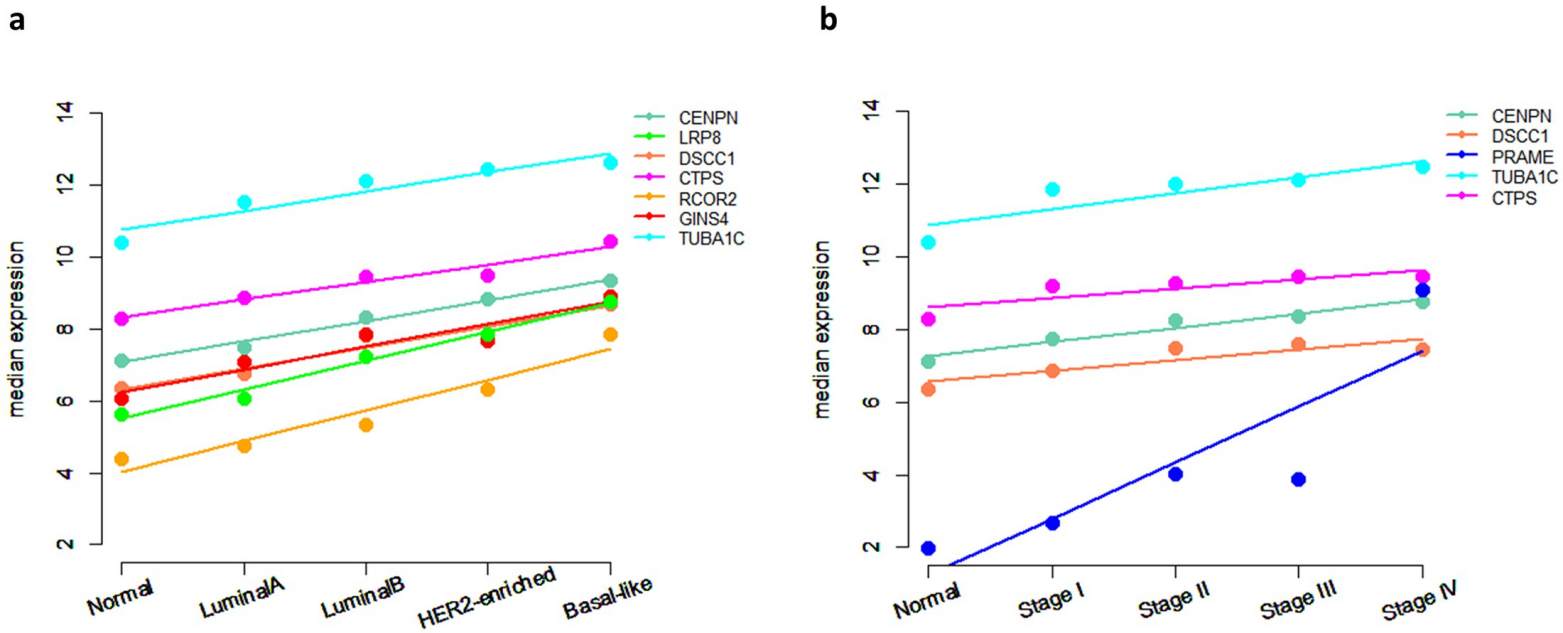
Linear regression model fitting. The median expression of the basal-like prognostic biomarkers is plotted against the phenotype varying from physiological to pathological condition (a) and against the pathological staging (b). Solid lines represent how the linear model fits the data. We showed, as a representative example, the results corresponding to the highest values of the model fitting index R-squared: R-squared (rounded to one decimal place) ≥ 0.9 for the subtype (a) and ≥ 0.7 for the staging (b).

To statistically quantify the increasing trend of the median expression values of these 11 switch genes as the phenotype varies from physiological to pathological condition passing across the different BC subtypes, we exploited a linear regression model, where the index R-squared estimates the goodness-of-fit. We found that all but one showed a very strong straight-line relationship (R-squared ≥ 0.7) between their median expression and the tumour subtypes (Table 1, RNA level), with the CENPN as the first on the list (R-squared = 0.99). These results were mostly confirmed by performing the same analysis using the pathological staging of the BC patients affected by PAM50 subtypes (Table 1, RNA level). Indeed, we observed that 6 basal-like specific switch genes (i.e., CENPN, DSCC1, CTPS, GINS4, TUBA1C, PRAME) reached an R-squared (rounded to one decimal place) ≥ 0.7 also with respect to the staging (Table 1, RNA level). The increasing trend of the top-ranked switch genes (highest R-squared) both with respect to the subtypes and the staging is depicted in Fig 4a and 4b, respectively.

**Fig 5:**
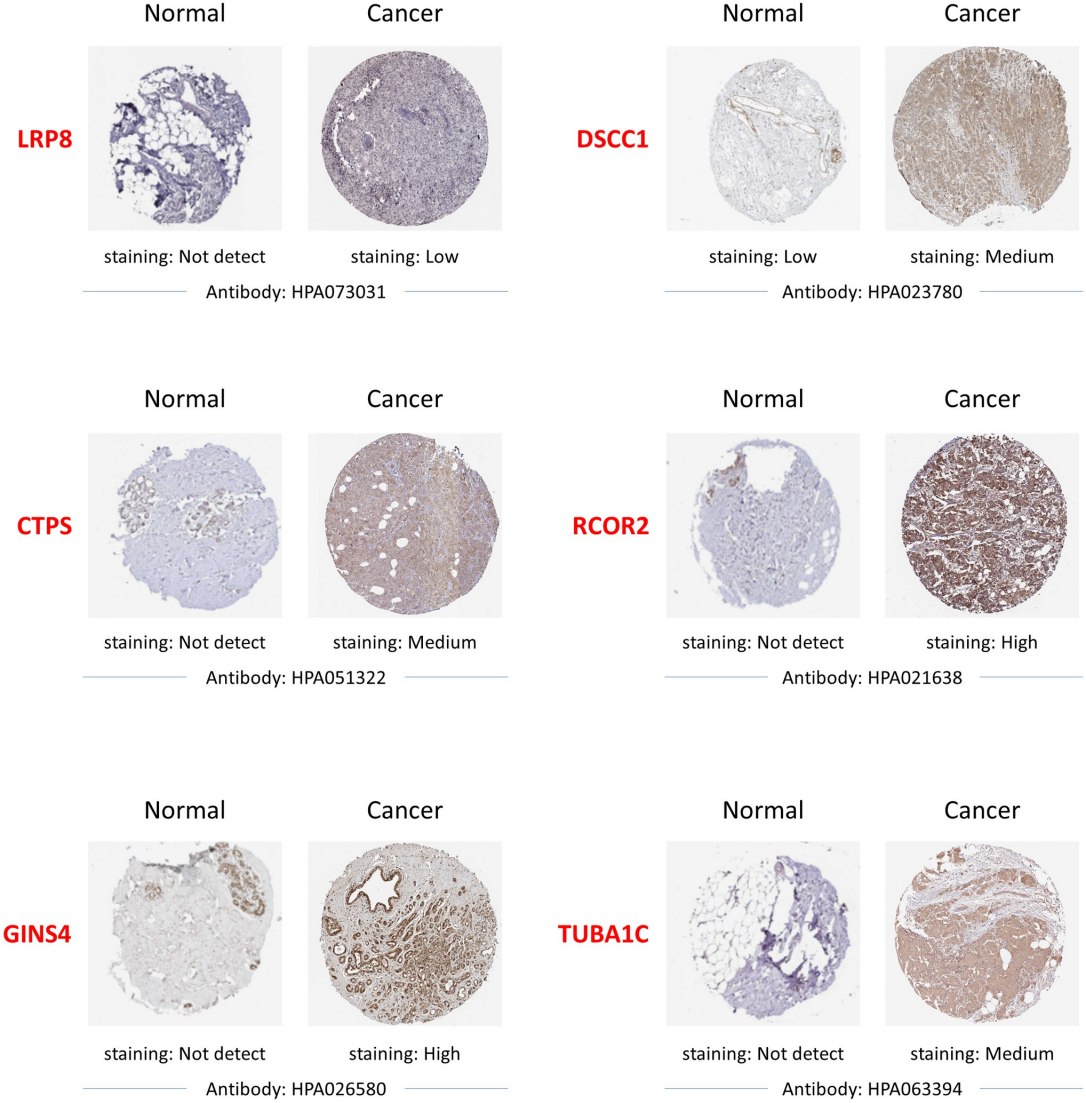
Immunohistochemistry results from the Human Protein Atlas. Representative immunohistochemistry images of the indicated switch genes in BC tissues and normal breast tissues obtained from the Human Protein Atlas.

In order to explore the expression patterns of the proteins encoded by the 11 prognostic switch genes, we queried the Human Protein Atlas (HPA) that provided representative immunohistochemistry images in BC tissues and normal breast tissues. As expected, we found that six of these proteins were overexpressed in BC tissues compared to normal breast tissues (Fig 5 and Table 1, Protein level). For the remaining ones, there are pending cancer and normal tissue analysis on the HPA and the immunohistochemistry images are not currently available (Table 1, Protein level).

### Gene regulatory network of the basal-like prognostic biomarkers

**Fig 6:**
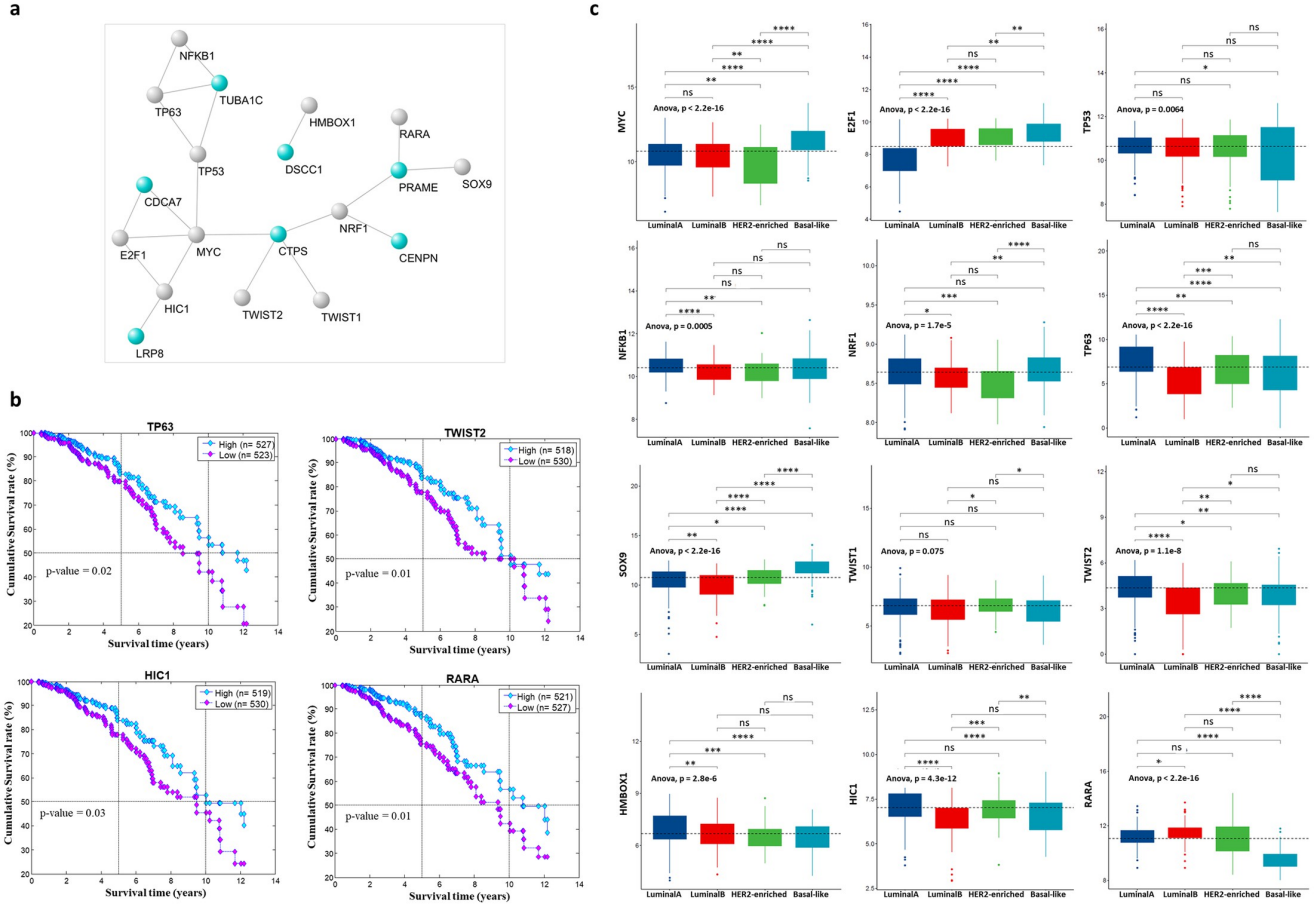
Gene regulatory network of the basal-like prognostic biomarkers. a) Network of the regulatory interactions among the identified switch genes and the known transcription factors (TFs). Light blue nodes represent switch genes; grey nodes represent transcription factors. b) TFs with a statistically significant prognostic value according to the Kaplan-Meier survival analysis. Kaplan-Meier analyzes to evaluate the correlations between the expression of the TFs and the OS in TCGA breast invasive carcinoma patients. Low- and high-expression groups refer to patients with expression levels lower and greater than the 50th percentile, respectively. c) Expression of the TFs in the gene regulatory network across the PAM50 breast cancer subtypes. The black dashed line reported in each plot indicates the median value used in the Kaplan-Meier survival analysis to split the low-expression and high-expression group. One-way ANOVA test was used to compare the means of the selected genes among the patients’ groups. T-test was used to perform multiple pairwise-comparisons and statistical significance was indicated by the star symbols (i.e., ns: p > 0.05, *: p ≤ 0.05, **: p ≤ 0.01, ***: p ≤ 0.001, ****: p ≤ 0.0001).

To provide some hints on which transcription factors (TFs) could regulate the expression of the 11 switch genes proposed as prognostic biomarkers for basal-like subtype, we built a gene regulatory network by combining information on both computationally predicted and experimentally validated TF-target relationships. In particular, we firstly exploited Pscan web tool [9] to predict TFs putatively able to bind the promoter regions of the selected switch genes. Then, we filtered the Pscan predictions keeping only the TFs known to physically interact with at least one switch genes in the human interactome [10]. These TF-target relationships were finally complemented with those experimentally validated from TRRUST database [11]. The final gene regulatory network was composed of seven switch genes and twelve TFs, including well-known TFs that, if deregulated, contribute to neoplastic transformation as MYC, TP53 and NFKB1 (Fig 6a and Table 1, DNA level). Interestingly, among the detected TFs, we found also four TFs (i.e., TP63, TWIST2, HIC1 and RARA) whose high expression appeared to be associated with the best prognosis for BC patients (Fig 6b). In accordance with this result, we observed that these four favourable TFs reached their highest value in the patients affected by the less aggressive BC subtype, i.e., luminal A (Fig 6c). It is worth noting that the other TFs of the gene regulatory network, in general, did not show a relevant increasing/decreasing trend across the different BC subtypes (Fig 6c), indicating that the overexpression of their target basal-like specific switch genes maybe not ascribed to their transcriptomic variations but rather to other genetic and/or epigenetic alterations.

### Genomic and epigenomic alterations of the basal-like prognostic biomarkers

Next, we investigated if the overexpression of the 11 basal-like prognostic biomarkers may depend on basal-like specific genomic alterations, such as Copy Number Variations (CNVs) and/or epigenomic alteration such as DNA methylation changes. In particular, we compared the CNVs and DNA methylation status of these 11 genes in basal-like subtype with respect to the less aggressive BC subtype, i.e., luminal A.

**Fig 7:**
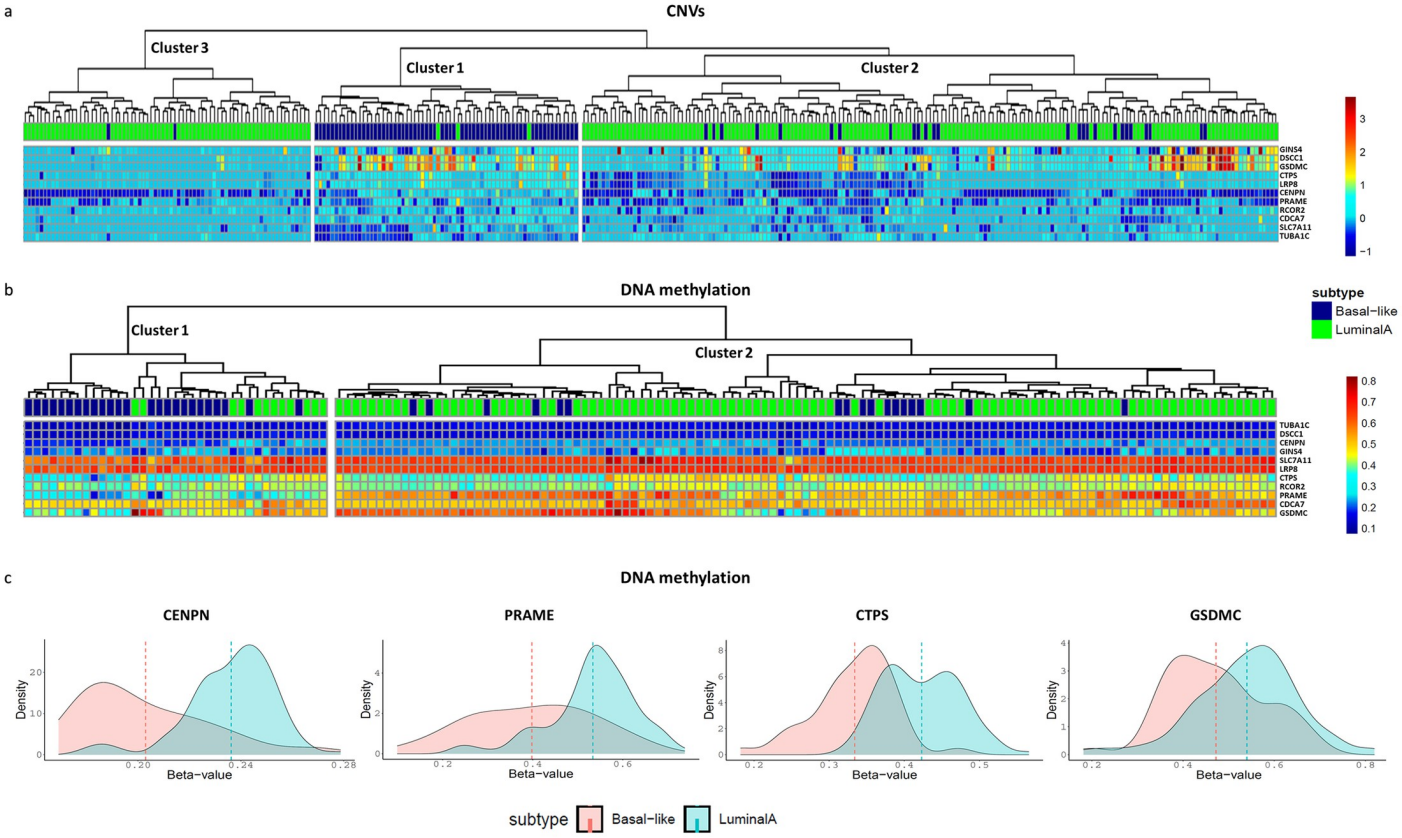
Genomic and epigenomic alterations of the basal-like prognostic biomarkers. a) Heatmap with dendrogram representing the unsupervised hierarchical clustering analysis based on CNVs data of TCGA-BRCA patients. The rows in the heatmap represent the 11 basal-like prognostic biomarkers. The columns correspond to basal-like and luminal A TCGA-BRCA patients: basal-like are indicated in dark blue and luminal A in green. The cells of the heatmap represent the log2 segment mean value of CNVs (ranging from -1 up to 3.5), for which colour code is indicated in the scale on the right-hand side of the figure. b) Heatmap with dendrogram representing the unsupervised hierarchical clustering analysis based on DNA methylation data of TCGA-BRCA patients. The rows in the heatmap represent the 11 basal-like prognostic biomarkers. The columns correspond to basal-like and luminal A TCGA-BRCA patients: basal-like are indicated in dark blue and luminal A in green. The cells of the heatmap represent beta-value (ranging from 0 to 1) extracted from Illumina 450k normalized data, for which colour code is indicated in the scale on the right-hand side of the figure. c) Distribution plot of beta-value of CENPN, GSDMC, PRAME and CTPS genes in basal-like and luminal A patients. Dashed lines represent the mean of beta-values for each patients’ group.

The CNVs analysis was performed on a total of 317 TCGA-BRCA patients (92 basal-like and 225 luminal A) for which CNVs data were available. Hierarchical clustering analysis on this data identified three main clusters and showed a different pattern of amplification and deletion in the selected genes between basal-like and luminal A patients (Fig 7a). Interestingly, Cluster 1 appears to be enriched in basal-like samples (64/67, 96%), whereas Cluster 2 (151/177, 85%) and Cluster 3 (71/73, 97%) are enriched in luminal A samples. Specifically, most of the basal-like patients belong to Cluster 1 (64/92, 70%; highlighted in dark blue in Fig 7a) and almost all luminal A belong to Cluster 2 and Cluster 3 (222/225, 99%; highlighted in green in Fig 7a). Cluster 1 features are mostly related to DSCC1, GSDMC amplifications (> 1 copy amplification per gene) along with TUBA1C deletion (>1 copy deletion per gene).

Aberrant DNA methylation is another epigenetic alteration that plays a fundamental role in precipitating the development of a large and diverse number of human cancers [12]. For this reason, we investigated a potential correlation between DNA methylation patterns and mRNA expression profiles of the 11 basal-like prognostic biomarkers in basal-like and luminal A patients. The DNA methylation data analysis was performed on a total of 152 TCGA-BRCA patients (37 basal-like and 152 luminal A) for which DNA methylation data were available. Hierarchical clustering analysis on this data identified two main clusters and showed a different DNA methylation status of the selected genes between basal-like and luminal A patients (Fig 7b). In particular, Cluster 1 is enriched in basal-like patients (25/37, 68%, highlighted in dark blue in Fig 7b) and could be associated with a low methylation level especially for CENPN, PRAME, GSDMC and CTPS genes (Table 1, DNA level). On the other hand, Cluster 2 is enriched in luminal A patients (98/115, 85%, highlighted in green in Fig 7b).

We compared the frequency of amplification and deletion events between basal-like and luminal A, using Fisher’s exact test (S2 Table) and we assessed the levels of methylation of the 11 basal-like prognostic biomarkers in the two groups (Fig 7c). We observed different scenarios of CNV alteration along with DNA methylation status of the 11 basal-like prognostic biomarkers. CTPS, CENPN and PRAME had a higher frequency of amplification events (> 1 copy amplification per gene) in basal-like, higher frequency of deletion events in luminal A group (p < 0.05, Fisher exact test) and they are hypomethylated in basal-like patients (Fig 7c). This first scenario showed the highest concordance between CNV alteration, DNA methylation levels and mRNA overexpression of these three genes in the basal-like group. Then, GSDMC is characterized by a higher frequency of amplification events in the basal-like group (p < 0.05, Fisher exact test) and is hypomethylated in basal-like patients (Fig 7c), probably overlapping with its mRNA overexpression in the basal-like group. LRP8 is more amplified in the basal-like group and more deleted in luminal A patients (p < 0.05, Fisher exact test), supporting a putative correlation with its mRNA overexpression in the basal-like group. DSCC1 and CDCA7 had a higher frequency of amplification in basal-like patients (p < 0.05, Fisher exact test), which could be correlated with their mRNA overexpression in that group. Difficult to place is the result of TUBA1C, as we found that this gene has a higher frequency of deletion events in basal-like compared to luminal A group.

## Discussion

BC is the neoplasia with the highest incidence and mortality affecting women worldwide [13] and is routinely analysed for ER, PR and HER2 using IHC-based assessment of protein expression levels and frequency [14]. This information is both prognostic and predictive, reflecting critical growth factor signalling dependencies that can be targeted for therapeutic benefit. Thanks to microarray technology, an intrinsic list of 496 genes used to classify BC into four molecular subtypes was identified [15, 16]. This makes BC a heterogeneous group of tumours that are diverse in behaviour, outcome, and response to therapy. Among the four intrinsic molecular subtypes (Luminal A, Luminal B, HER2 positive and Basal-like), the basal-like has the worst prognosis as often aggressive and highly recurrent lesions. Basal-like subtype lacks expression of the ER, PR, and HER2 [17] and histologically shows a high grade, high mitotic indices, presence of central necrotic or fibrotic zones, pushing borders of invasion, lymphocytic infiltrate and atypical medullary features [18]. These features limit therapeutic response and impact the refractory nature of these tumours [19], thus, basal-like patients have a poor prognosis and short-term disease-free (DFS) and OS. Then, finding key genes associated with basal-like subtype aggressiveness would help identify prognostic biomarkers for survivals of BC patients as well as the most suitable target genes for new anticancer treatments [20].

Thanks to large international consortia such as The Cancer Genome Atlas (TCGA) [15] and the International Cancer Genome Consortium (ICGC) [21, 22], significant inroads have been made characterizing the genomic diversity of BC using next-generation sequencing of RNA and DNA from human clinical samples. SWIM is a novel promising tool that builds upon the structural properties of gene co-expression networks to unveil key genes (called switch genes) likely associated with drastic physiological changes in many biological settings [23, 24]. Until now, the relevance of switch genes related to an observed phenotype has been widely assessed through several applications [2, 23, 25–29]. In particular, recently in [2], by using the transcriptomic profiling of TCGA breast collection [30], we analysed a total of 505 subjects for which PAM50 subtypes were provided (229 Luminal A, 120 Luminal B, 58 HER2-enriched, and 98 Basal-like) and compared their expression profiles with those of normal samples to identify switch genes associated with the transition between normal condition and each BC subtype. From this comparative analysis, we found both switch genes shared among four subtypes and switch genes specific for each subtype. In the study carried out in [2], we focused on the common switch genes and performed several in silico analysis and *in vitro* and *ex vivo* experiments to highlight molecular signatures shared among all BC subtypes. However, we believe that the in-depth investigation of the subtype-specific switch genes can allow us to find novel putative associations between gene functionality and subtype-specific aggressiveness especially for more aggressive BC subtypes. So, the goal of this study was to identify among the switch genes specific for basal-like subtype, those linked to a poor prognosis. In the wake of our recent study [2], the 108 switch genes found to be basal-like specific have been analysed for their prognostic abilities, and among them, 15 shown a significant prognostic role as demonstrated by Kaplan-Maier curves results. Of these, 11 appeared to be unfavourable prognostic genes (i.e., CTPS, CDCA7, GSDMC, LRP8, TUBA1C, CENPN, PRAME, SLC7A11, GINS4, DSCC1, RCOR2) as their overexpression was found to be associated with poorer OS; this result was confirmed using another BC dataset collection (http://kmplot.com/analysis/). Interestingly, these 11 switch genes showed their highest mRNA overexpression in the basal-like compared to the other BC subtypes, and this data further strengthens the hypothesis that these switch genes could be poor prognostic biomarkers in basal-like subtype affected patients (Fig 3). After that, by a linear regression model, we found a straight-line relationship (from 0.7 up to 0.99) among CENPN, LRP8, DSCC1, CTPS, RCOR2, GINSS4, TUBA1C and PRAME with tumour subtypes and staging, while SLC7A11 and CDCA7 correlated only with subtypes. No correlation between GSDMC with subtypes and staging were found. The protein levels of these 11 switches in BC specimens were evaluated by querying the Human Protein Atlas. IHC results were examined confirming that 6 (CTPS, LRP8, TUBA1C, DSCC1, GINS4, RCOR2) of the 11 proteins were overexpressed in BC tissues compared to normal ones. For the remaining proteins, IHC results were not yet available in the Human Protein Atlas (CDCA7, GSDMC, SLC7A11, PRAME and CENPN), nevertheless, the above citations confirmed us that all these switch proteins were overexpressed both in BC cell lines and tissues. These results led us to suspect their role in the neoplastic transformation. In fact, data from the literature, follow detailed, give to these molecules a tumorigenic characteristics being found deregulated in different human cancers including TNBC subtype, so as to make more robust our findings.

CTPS1 (CTP synthase 1) gene, encodes an enzyme responsible for the catalytic conversion of UTP (uridine triphosphate) to CTP (cytidine triphosphate). This reaction is an important step in the biosynthesis of phospholipids and nucleic acids. Increased levels of the protein have been linked to several mammalian cancer types such as sarcoma [31], hepatoma [32, 33] and leukaemia [33], where the activity of this enzyme is both transformations- and progression-linked, marking out this enzyme as an important target in the design of chemotherapy. More important, *in vitro* experiments performed on BC cell lines demonstrated that CTP depletion results in a senescence-like growth arrest through activation of p53, whereas cells with mutated p53 undergo differentiation or apoptotic cell death [34].

LRP8 (LDL receptor-related protein 8) gene, encodes a member of the low-density lipoprotein receptor (LDLR) family. A recent study demonstrated that LRP8 was more strongly expressed in BC without hormone receptor expression (TNBC and HER2 positive) than in luminal tumours (Luminal A and Luminal B) [35]. Authors found that LRP8 depletion promoted apoptosis, impaired cell proliferation and colony formation suggesting that LRP8 has tumourigenic properties. These findings were further confirmed by experiments showing that LRP8 depletion slowed tumour growth in an *in vivo* xenograft model. Moreover, inhibition of LRP8 was found to attenuate Wnt/β-catenin signalling to suppress breast cancer stem cells (BCSCs) enriched in TNBC and responsible for chemoresistance and metastasis [35, 36].

Tubulin alpha-1C chain is a protein that in humans is encoded by the TUBA1C gene. TUBA1C is a member of the tubulin families and several studies demonstrated that its upregulation promotes oncogenesis and predicts poor prognosis in different tumour types [37, 38]. TUBA1C, TUBA1B and the β-tubulin isoform TUBB were found as isoforms with the highest expression levels compared to other isoforms in BC cell lines, and TUBA1C and TUBB were overexpressed in BC tumours compared to the normal breast tissues [39]. Also, the prognostic role of TUBA1C as a marker linked to the progression of BC was highlighted by [40], it was associated with lower OS in BC patients [41], and GTSE1 and TUBA1C combined predicted 100% probability of developing TNBC in whites [42].

Recently, overexpression of DSCC1 (DNA replication and sister chromatid cohesion 1) was found to increase proliferation, invasion and migration of breast carcinoma cells, as well as its knockdown showed opposite outcomes [43, 44]. Besides, the authors found that DSCC1 could promote breast carcinoma progression by activating the Wnt/β-catenin signalling and inhibiting p53 protein.

PRAME nuclear receptor transcriptional regulator gene encodes an antigen that is preferentially expressed in human melanomas. The approved mutual link between BC and melanoma conditions emphasized the idea of utilizing this marker for targeting BC progression as well. Indeed, this protein was found to be involved in BC growth and metastasis and promote epithelial-to-mesenchymal transition in TNBC [45–47], suggesting that PRAME could serve as a prognostic biomarker and/or therapeutic target in TNBC.

Cancer cell requires excess nutrients to meet their biosynthetic and bioenergetics needs and to maintain appropriate redox balance. Glucose and glutamine are important nutrients supporting cancer cell survival. SLC7A11 (solute carrier family 7 member 11) gene encodes a member of a heteromeric, sodium-independent, anionic amino acid transport system that is highly specific for cysteine and glutamate; imports extracellular cystine coupled to the efflux of intracellular glutamate. SLC7A11 expression can be induced under various stress conditions, likely as an adaptive response to enable cells to restore redox homeostasis and maintain survival under stress conditions [48]. The upregulation of SLC7A11 was found correlated with a poor response to treatment in different cancers including breast [49]. Recent evidence support that cancer cells upregulate SLC7A11 expression through diverse mechanisms to enhance their antioxidant defence and to suppress ferroptosis, a key tumour suppression mechanism [50].

The gasdermin (GSDM) superfamily consist of several molecules involved in cell pyroptosis. Recently, various studies have revealed the dysfunction and abnormal expression of the GSDM family in multiple human cancers, implying the potential roles in tumorigenesis. GSDMC (gasdermin C), a member of GSDM superfamily was found to promote cell proliferation in colorectal cancer [51], and high expression of GSDMC in BC [52] and lung adenocarcinoma [53] correlates with poor survival.

CDCA7 (cell division cycle associated 7), was found to be elevated in various types of human cancer, including colon, lung, prostate and breast cancers [54], suggesting that this protein might play an important role in the development of cancer. Interestingly, CDCA7 is a DNA-binding protein and regulates the gene expression of the tumour-promoting effect of c-Myc and E2F1. Recently the role of CDCA7 in TNBC subtype has been partially clarified and authors found that high expression of CDCA7 was associated with metastatic relapse status and predicted poorer disease-free survival in patients with TNBC via transcriptionally upregulating the expression of EZH2 [55].

Centromere proteins (CENPs), which comprise 18 subtypes, are related dynamically to association and dissociation during mitosis with microtubule regulation. Among the CNPs, the protein encoded by CENPN (centromere protein N) gene, binds directly to the centromere-targeting domain of CENP-A. CENP-N depletion causes down-regulation of several CENPs and is considered essential for making a new centromere. Other functions of CENP-N, including its deregulation in BC are unclear, except the study that associated elevated expression of this protein with significantly increased mortality and risk of recurrence in BC smokers in contrast with non-smokers BC subjects [56].

RCOR2 (REST corepressor 2) is a protein-coding gene. Gene Ontology (GO) annotations related to this gene include DNA-binding transcription factor activity and transcription corepressor activity. To date, its involvement in the growth and progression of BC was not yet bee investigated.

GINS4 is a subunit of the GINS complex (GINS1, GINS2, GINS3, and GINS4 subunits) involved in the initiation and progression of DNA replication [57]. GINS4 was found highly expressed in lung, bladder and colorectal cancers, and its downregulation in the bladder and colorectal cancers inhibits growth and cell cycle and accelerate cell apoptosis progression *in vitro* as well as inhibits tumorigenesis *in vivo* [58, 59]. As for RCOR2 protein, GINS4 involvement in the growth and progression of BC was not yet bee investigated.

Based on these findings, we felt compelled to understand which regulatory events might be responsible for their upregulation in basal-like subtype. So, we investigated whether the deregulated expression of the selected switch genes could be related to the activity of known transcription factors, copy number variation and DNA methylation. The construction of a gene regulatory network showed how these switch genes interact with several TFs known to be altered in cancer condition (MYC, TP53 and NFKB1), including in TNBC [60–62]. Nevertheless, we did not expect, but we were not surprised, that some of the identified TFs (TP63, TWIST2, HIC1 and RARA) were overexpressed in luminal A rather than in basal-like patients. So, being found also linked to a better prognosis, these results bring us to the hypothesis that these TFs could not be involved in the basal-like switch genes activation. Interestingly, we found that for most of the 11 switch genes their overexpression seems to be ascribed to genetic and/or epigenetic alterations. Indeed, we found that CTPS, CENPN, PRAME and GSDMC were found both hypomethylated and amplified in basal-like subtype as well as, except for GSDMC, also deleted in luminal A subtype; together these results are strongly in line with their expression data alterations found in the basal-like subtype. In the same way, also DSCC1 and CDCA7 were found amplified in basal-like, and CNVs profiles analysis demonstrated that the copy number amplification of two switch genes, DSCC1 and GSDMC, clustered for basal-like patients. Results on TUBA1C were somewhat controversial as this gene was found to be amplified in luminal A subtype and no genetic or epigenetic changes were found in basal-like subtype; for this switch gene seems that neither amplification nor methylation status is responsible for its overexpression in the basal-like subtype. Taken together these data enrich the pathophysiological and prognostic role of these genes in BC basal-like subtype.

## Limitations of the study

The first limitation of this study is that it is based on gene expression data and, it would need further deepening at the protein level as soon as proteomic data will be available on large scale for the disease covered in this analysis. However, even if the cause-effect relationship cannot be directly inferred by expression data, correlation networks may highlight disease co-modulated genes that are functionally coordinated in response to an external stimulus, implying that they may be part of the same complexes or pathways, and may influence each other or maybe influenced by the same underlying mechanism(s).

A further limitation of this study is that our entire results should be validated by using another independent dataset. However, the proposed bioinformatics pipeline requires a huge quantity of transcriptomic, genomic, epigenomic, and clinical data related to patients affected by different breast cancer subtypes and, currently, TCGA is the only free repository providing simultaneously all this information for the same patient cohort.

Lastly, it would have been very interesting to correlate the expression of the 11 genes constituting the basal-like gene signature with the Ki-67 labeling index (Ki-67LI), defined as the percentage of Ki-67 antigen positive cells. Indeed, Ki-67LI is commonly used as proliferation marker and it has frequently been associated with the clinical outcome of TNBC patients [63, 64]. Unfortunately, TCGA does not provide this index among the clinical data of the patients affected by breast cancer.

## Conclusions

In conclusion, our study showed that 11 basal-like specific switch genes are overexpressed in BC tissues compared to normal counterpart and associated with BC patients prognosis acting as unfavourable prognostic markers. Also, their highest expression was found in the basal-like subtype and this overexpression could be putatively related to genetic and epigenetic alterations as well as the action of important transcription factors. Taken together, these results turn on a beam of light on CTPS, CDCA7, GSDMC, LRP8, TUBA1C, CENPN, PRAME, SLC7A11, GINS4, DSCC1 and RCOR2 that can constituite a gene signature to evaluate the prognosis of basal-like breast cancer patients independently from the therapeutic intervention. It is worth to stress that our study has a purely computational nature and experimental validations would be necessary to investigate the actual role of the identified genes in the framework of basal-like breast cancer. However, we belive that our findings could provide advancements in the ongoing effort to identify specific prognostic biomarkers for basal-like subtype in order to improve the clinical management of this disease.

## Methods

### The Cancer Genome Atlas

The Cancer Genome Atlas (TCGA) is a comprehensive project born in 2006 from the joint effort between the National Cancer Institute and the National Human Genome Research Institute to improve diagnosis methods and treatments against cancers [30]. This project molecularly characterized over 20,000 primary cancer and matched normal samples spanning 33 cancer types and, in the last years, generated over 2.5 petabytes of genomic, epigenomic, transcriptomic, and proteomic data. All this data, which has already lead to improvements in our ability to diagnose, treat, and prevent cancer, will remain publicly available for anyone in the research community to use. In this study, we exploited TCGA to obtain transcriptomic, clinicopathological, Copy Number Variations (CNVs) data referring to patients affected by breast invasive carcinoma. Male samples, as well as samples undergoing a neoadjuvant treatment, were removed from the cohort under study.

### The Human Protein Atlas

The Human Protein Atlas is a research program initiated in 2003 to map all the human proteins in cells, tissues and organs using an integration of various omics technologies, including antibody-based imaging, mass spectrometry-based proteomics, transcriptomics and systems biology [65]. All the data in the knowledge resource is open access to allow scientists both in academia and industry to freely access the data for exploration of the human proteome. In this study, the Human Protein Atlas website (https://www.proteinatlas.org) was leveraged to identify tumour-type specific proteins expression patterns and to perform immunohistochemistry image a direct comparison of the protein expression of selected prognostic indicators between normal and tumour breast tissues.

### SWIM software

SWIM (SWitchMiner) is a new methodology that considers differentially expressed genes within the co-expression network framework to predict important genes affected by a disease of interest, and combines this information with a structured network of correlated patterns. Considering the topological properties of the nodes and assessing their functional roles according to their ability to convey information within and between modules in the network, SWIM identifies a small pool of genes (known as switch genes) that are associated with intriguing patterns of molecular co-abundance and play a crucial role in the observed phenotype (transitions) [23]. SWIM is a freely available software developed in MATLAB that implements a series of well-defined steps described in details in the Supplementary Information of [23]. Up to now, SWIM has sparked a widespread interest within the scientific community thanks to the promising results obtained through its application in a broad range of phenotype-specific scenarios, spanning from complex diseases to grapevine berry maturation [23, 25–28, 66].

### Kaplan-Meier survival analysis

To analyze the correlation between the expression level of the 108 basal-like specific switch genes and patient overall survival (OS) and therefore to evaluate their prognostic value, we used the RNA-sequencing data from TCGA to split the entire cohort of BC patients (1049 samples) into two groups (called low-expression and high-expression group) according to the upper and lower expression quartile. In particular, low- and high-expression groups refer to patients with expression levels of the given switch gene lower and greater than the 50th percentile (i.e., median), respectively. For each patient cohort, the cumulative survival rates were computed for each switch gene according to the Kaplan-Meier (KM) method [67] on the clinical metadata provided by TCGA. For each switch gene, the survival outcomes of the two patients groups were compared by the log-rank test. Switch genes with log-rank p-values less than 0.05 were suggested as candidate prognostic biomarkers. In particular, the lower the p-value, the better the separation between the two prognosis groups. If the group of patients with high expression of the selected prognostic gene has a higher observed event than expected event (worst prognosis), it is defined as an unfavourable prognostic gene; otherwise, if its high expression is associated with the best prognosis, it is a favourable prognostic gene.

To confirm the prognostic value of the basal-like specific switch genes points out from the KM survival analysis on the TCGA breast invasive carcinoma patients, we performed the KM analysis on different breast cancer dataset. To do this, we exploited the Kaplan-Meier plotter website (http://kmplot.com/analysis/), which integrates gene expression data and OS information downloaded from GEO, EGA and TCGA for several types of cancer [68]. We ran Kaplan-Meier plotter by considering the entire breast cancer database including 7,830 unique samples from 55 independent affymetrix datasets [69] and by dividing patients into high and low expression group based on the auto selected best cuttoff computed between the lower and upper quartiles of switch genes expression.

### Statistical methods

The one-way analysis of variance (ANOVA) is an extension of independent two-samples t-test for comparing means in a situation where there are more than two groups. In one-way ANOVA, the data is organized into several groups based on one single grouping variable (also called factor variable). In this study, the one-way ANOVA test was used to compare the means of selected genes in patients grouped based on the PAM50 breast cancer subtypes. A p-value ≤ 0.05 indicated that at least two groups significantly differ from each other and multiple pairwise-comparisons exploiting the t-test method were performed to identify which ones.

### Gene regulatory network

The gene regulatory network of the selected switch genes was constructed by integrating information from Pscan [9], TRRUST [11] and the human interactome (i.e., that is the network of all physical interactions within a cell, from protein-protein to regulatory protein–DNA and metabolic interactions [70]).

Pscan is a web tool designed to computationally predict TF-target regulatory relationships [9]. In particular, it scans the sequence of the promoter regions from an input gene list with motifs describing the binding specificity of known transcription factors and assesses which motifs are significantly over-or under-represented, suggesting which transcription factors could be common regulators of the input genes. In this study, the promoter regions were identified as the genomic regions spanning from -450 to +50 nucleotides to transcription start sites and the TF binding profiles were retrieved from JASPAR 2018 database [71].

TRRUST is a freely available and manually curated database containing 8,444 TF-target regulatory relationships of 800 human transcription factors. These relationships have been derived from PubMed articles describing small-scale experimental studies of transcriptional regulations by using a sentence-based text mining approach [11].

The human interactome, also called protein-protein interaction (PPI) network, was downloaded from Cheng and coauthors [10], where the authors assembled their in-house systematic human interactome with 15 commonly used databases with several types of experimental evidences (e.g., binary PPIs from three-dimensional protein structures; literature-curated PPIs identified by affinity purification followed by mass spectrometry, Y2H, and/or literature-derived low-throughput experiments; signalling networks from literature-derived low-throughput experiments; kinase-substrate interactions from literature-derived low-throughput and high-throughput experiments). This version of the interactome is composed of 217,160 protein-protein interactions connecting 15,970 unique proteins.

### Copy Number Variations (CNVs) data analysis

Copy Number Variations (CNVs) data of TCGA-BRCA project were retrieved from TCGA repository and reported contiguous chromosome regions with log2 ratio segment means in a tab-delimited format. To obtain segment means values of CNVs of the selected genes for the enrolled patients, we employed GISTIC 2.0 software [72]. Gistic’s parameters used in this study are the following:

-*b “path file*; -*seg “filename”*; -*refgene refgenefiles/hg19*.*UCSC*.*add_miR*.*140312*.*refgene*.*mat*; -*mk genome*.*info*.*6*.*0_hg19*.*na31_minus_frequent_nan_probes_sorted_2*.*1*.*txt*; -*maxspace 2000*; -*ta 0*.*3*; -*td 0*.*3*; -*js 4*;-*qvt 0*.*01*; -*conf 0*.*99*; -*genegistic 1*; -*armpeel 1*; -*savegene 1*.

The hierarchical clustering analysis was performed by using “Canberra” as clustering distance and “ward.D2” as clustering method. The association between the CNVs status of the selected genes and the BC subtypes was evaluated using Fisher’s exact test.

### DNA methylation data analysis

DNA methylation data of TCGA-BRCA project were retrieved by Firehorse TCGA GDAC browser (https://gdac.broadinstitute.org/). The methylation data were acquired by the Illumina 450K array, which measures the level of methylation as a beta value for more than 450 000 CpG sites on the Illumina chip. The data contained information for about 485 578 CpG sites. To make available and pre-process methylation data in R environment, we used minfi package [73]. Pre-processing was performed using an in-house R scripts that eliminated probes with no methylation level detectable, removed all known single-nucleotide polymorphism (SNP)-associated CpG sites, associated CpG sites with known genes and matched patients and genes selected in our study. The hierarchical clustering analysis was performed by using “Euclidean” as clustering distance and “ward.D2” as clustering method.

## Supporting information

S1 Fig Switch genes with a favourable prognostic value from the survival analysis on TCGA data. Kaplan-Meier analyzes to evaluate the correlations between the expression of the basal-like specific switch genes and the OS in TCGA breast invasive carcinoma patients. Low- and high-expression groups refer to patients with expression levels lower and greater than the 50th percentile, respectively.
(PNG)

S2 Fig Expression of the switch genes with an unfavourable prognostic value in TCGA basal-like tumour tissues and adjacent normal tissues. Gene expression levels of the 11 basal-like specific switch genes point out from the Kaplan-Meier survival analysis in basal-like and normal samples available from TCGA repository. T-test was used to compare the means of the selected genes between the two sample groups (Normal and Tumour) and statistical significance was indicated by the star symbols (i.e., ns: p > 0.05, *: p ≤ 0.05, **: p ≤ 0.01, ***: p ≤ 0.001, ****: p ≤ 0.0001).
(PNG)

S1 Table Basal-like specific switch genes. The table is composed of two separated sheets. The first sheet reports the complete list of 108 switch genes found to be specific for basal-like breast cancer subtype. The second sheet reports the complete list of 11 switch gene whose activation was found to be associated with the worst prognosis from the KM survival analysis on the TCGA breast invasive carcinoma patients.
(XLSX)

S2 Table CNVs results. The table reports the results of the CNVs data analysis for the 11 basal-like prognostic biomarkers.
(XLSX)

## References

[1] Ferlay J., et al., Estimating the global cancer incidence and mortality in 2018: GLOBOCAN sources and methods. International Journal of Cancer, 2019. 144(8): p. 1941–1953. doi: 10.1002/ijc.31937 30350310

[2] Grimaldi A.M., et al., The New Paradigm of Network Medicine to Analyze Breast Cancer Phenotypes. International Journal of Molecular Sciences, 2020. 21(18). doi: 10.3390/ijms21186690 32932728 PMC7555916

[3] Gazinska P., et al., Comparison of basal-like triple-negative breast cancer defined by morphology, immunohistochemistry and transcriptional profiles. Modern Pathology, 2013. 26(7): p. 955–966. doi: 10.1038/modpathol.2012.244 23392436

[4] Lin N.U., et al., Clinicopathologic Features, Patterns of Recurrence, and Survival Among Women With Triple-Negative Breast Cancer in the National Comprehensive Cancer Network. Cancer, 2012. 118(22): p. 5463–5472. doi: 10.1002/cncr.27581 22544643 PMC3611659

[5] Badve S., et al., Basal-like and triple-negative breast cancers: a critical review with an emphasis on the implications for pathologists and oncologists. Modern Pathology, 2011. 24(2): p. 157–167. doi: 10.1038/modpathol.2010.200 21076464

[6] Milioli H.H., et al., Basal-like breast cancer: molecular profiles, clinical features and survival outcomes. Bmc Medical Genomics, 2017. 10. doi: 10.1186/s12920-017-0250-9 28351365 PMC5370447

[7] Toft D.J. and Cryns V.L., Minireview: Basal-Like Breast Cancer: From Molecular Profiles to Targeted Therapies. Molecular Endocrinology, 2011. 25(2): p. 199–211. doi: 10.1210/me.2010-0164 20861225 PMC3035993

[8] Gyoerffy B., et al., An online survival analysis tool to rapidly assess the effect of 22,277 genes on breast cancer prognosis using microarray data of 1,809 patients. Breast Cancer Research and Treatment, 2010. 123(3): p. 725–731. doi: 10.1007/s10549-009-0674-9 20020197

[9] Zambelli F., Pesole G., and Pavesi G., Pscan: finding over-represented transcription factor binding site motifs in sequences from co-regulated or co-expressed genes. Nucleic Acids Research, 2009. 37: p. W247–W252. doi: 10.1093/nar/gkp464 19487240 PMC2703934

[10] Cheng F., et al., Network-based approach to prediction and population-based validation of in silico drug repurposing. Nature Communications, 2018. 9. doi: 10.1038/s41467-018-05116-5 30002366 PMC6043492

[11] Han H., et al., TRRUST v2: an expanded reference database of human and mouse transcriptional regulatory interactions. Nucleic Acids Research, 2018. 46(D1): p. D380–D386. doi: 10.1093/nar/gkx1013 29087512 PMC5753191

[12] Klutstein M., et al., DNA Methylation in Cancer and Aging. Cancer Research, 2016. 76(12): p. 3446–3450. doi: 10.1158/0008-5472.CAN-15-3278 27256564

[13] Bray F., et al., Global cancer statistics 2018: GLOBOCAN estimates of incidence and mortality worldwide for 36 cancers in 185 countries. Ca-a Cancer Journal for Clinicians, 2018. 68(6): p. 394–424. doi: 10.3322/caac.21492 30207593

[14] Goldhirsch A., et al., Personalizing the treatment of women with early breast cancer: highlights of the St Gallen International Expert Consensus on the Primary Therapy of Early Breast Cancer 2013. Annals of Oncology, 2013. 24(9): p. 2206–2223. doi: 10.1093/annonc/mdt303 23917950 PMC3755334

[15] Koboldt D.C., et al., Comprehensive molecular portraits of human breast tumours. Nature, 2012. 490(7418): p. 61–70. doi: 10.1038/nature11412 23000897 PMC3465532

[16] Parker J.S., et al., Supervised Risk Predictor of Breast Cancer Based on Intrinsic Subtypes. Journal of Clinical Oncology, 2009. 27(8): p. 1160–1167. doi: 10.1200/JCO.2008.18.1370 19204204 PMC2667820

[17] Prat A., et al., Molecular Characterization of Basal-Like and Non-Basal-Like Triple-Negative Breast Cancer. Oncologist, 2013. 18(2): p. 123–133. doi: 10.1634/theoncologist.2012-0397 23404817 PMC3579595

[18] Putti T.C., et al., Estrogen receptor-negative breast carcinomas: a review of morphology and immunophenotypical analysis. Modern Pathology, 2005. 18(1): p. 26–35. doi: 10.1038/modpathol.3800255 15332092

[19] Rakha E.A., Reis-Filho J.S., and Ellis I.O., Impact of basal-like breast carcinoma determination for a more specific therapy. Pathobiology, 2008. 75(2): p. 95–103. doi: 10.1159/000123847 18544964

[20] Grimaldi A.M. and Incoronato M., miRNA-based Therapeutics in Breast Cancer: A Systematic Review. Frontiers in Oncology, 2021. 11: p. 1472. doi: 10.3389/fonc.2021.668464 34026646 PMC8131824

[21] Stephens P.J., et al., The landscape of cancer genes and mutational processes in breast cancer. Nature, 2012. 486(7403): p. 400-+. doi: 10.1038/nature11017 22722201 PMC3428862

[22] Zhang J., et al., International Cancer Genome Consortium Data Portal—a one-stop shop for cancer genomics data. Database, 2011. 2011. doi: 10.1093/database/bar026 21930502 PMC3263593

[23] Paci P., et al., SWIM: a computational tool to unveiling crucial nodes in complex biological networks. Scientific Reports, 2017. 7. 10.1038/srep44797 PMC5357943 28317894

[24] Paci P., et al., Gene co-expression in the interactome: moving from correlation toward causation via an integrated approach to disease module discovery. NPJ systems biology and applications, 2021. 7(1): p. 3–3. doi: 10.1038/s41540-020-00168-0 33479222 PMC7819998

[25] Palumbo M.C., et al., Integrated Network Analysis Identifies Fight-Club Nodes as a Class of Hubs Encompassing Key Putative Switch Genes That Induce Major Transcriptome Reprogramming during Grapevine Development. Plant Cell, 2014. 26(12): p. 4617–4635. doi: 10.1105/tpc.114.133710 25490918 PMC4311215

[26] Fiscon G., et al., Computational identification of specific genes for glioblastoma stem-like cells identity. Scientific Reports, 2018. 8. 10.1038/s41598-018-26081-5 PMC5958093 29773872

[27] Fiscon G., Conte F., and Paci P., SWIM tool application to expression data of glioblastoma stem-like cell lines, corresponding primary tumors and conventional glioma cell lines. Bmc Bioinformatics, 2018. 19. doi: 10.1186/s12859-018-2421-x 30497369 PMC6266956

[28] Falcone R., et al., BRAF(V600E)-mutant cancers display a variety of networks by SWIM analysis: prediction of vemurafenib clinical response. Endocrine, 2019. 64(2): p. 406–413. doi: 10.1007/s12020-019-01890-4 30850937

[29] Fiscon G., et al., Gene network analysis using SWIM reveals interplay between the transcription factor-encoding genes HMGA1, FOXM1, and MYBL2 in triple-negative breast cancer. Febs Letters, 2021. 10.1002/1873-3468.14085 33835503

[30] Weinstein J.N., et al., The Cancer Genome Atlas Pan-Cancer analysis project. Nature Genetics, 2013. 45(10): p. 1113–1120. doi: 10.1038/ng.2764 24071849 PMC3919969

[31] Weber G., et al., PURINE AND PYRIMIDINE ENZYMIC PROGRAMS AND NUCLEOTIDE PATTERN IN SARCOMA. Cancer Research, 1983. 43(3): p. 1019–1023. 6825077

[32] Kizaki H., et al., Increased cytidine 5’-triphosphate synthetase activity in rat and human tumors. Cancer research, 1980. 40(11): p. 3921–7. 7471043

[33] Williams J.C., et al., Increased CTP synthetase activity in cancer cells. Nature, 1978. 271(5640): p. 71–3. doi: 10.1038/271071a0 203856

[34] Huang M., et al., Cyclopentenyl Cytosine Induces Senescence in Breast Cancer Cells through the Nucleolar Stress Response and Activation of p53. Molecular Pharmacology, 2011. 80(1): p. 40–48. doi: 10.1124/mol.110.070284 21464199 PMC3127532

[35] Maire V., et al., LRP8 is overexpressed in estrogen-negative breast cancers and a potential target for these tumors. Cancer Medicine, 2019. 8(1): p. 325–336. doi: 10.1002/cam4.1923 30575334 PMC6346259

[36] Lin C.-C., et al., Targeting LRP8 inhibits breast cancer stem cells in triple-negative breast cancer. Cancer Letters, 2018. 438: p. 165–173. doi: 10.1016/j.canlet.2018.09.022 30227220 PMC6945120

[37] Wang J., et al., Oncogene TUBA1C promotes migration and proliferation in hepatocellular carcinoma and predicts a poor prognosis. Oncotarget, 2017. 8(56): p. 96215–96224. doi: 10.18632/oncotarget.21894 29221200 PMC5707094

[38] Albande M.A.H., et al., Upregulated Expression of TUBA1C Predicts Poor Prognosis and Promotes Oncogenesis in Pancreatic Ductal Adenocarcinoma via Regulating the Cell Cycle. Frontiers in Oncology, 2020. 10. 10.3389/fonc.2020.00049 PMC7033491 32117719

[39] Nami B. and Wang Z., Genetics and Expression Profile of the Tubulin Gene Superfamily in Breast Cancer Subtypes and Its Relation to Taxane Resistance. Cancers, 2018. 10(8). doi: 10.3390/cancers10080274 30126203 PMC6116153

[40] Wang C.C.N., et al., Identification of Prognostic Candidate Genes in Breast Cancer by Integrated Bioinformatic Analysis. Journal of Clinical Medicine, 2019. 8(8). doi: 10.3390/jcm8081160 31382519 PMC6723760

[41] Chen D., et al., SEMA6D Expression and Patient Survival in Breast Invasive Carcinoma. International Journal of Breast Cancer, 2015. 2015. doi: 10.1155/2015/539721 25973277 PMC4417987

[42] Ramos J., et al., Sensitivity to differential NRF1 gene signatures contributes to breast cancer disparities. Journal of Cancer Research and Clinical Oncology, 2020. 146(11): p. 2777–2815. doi: 10.1007/s00432-020-03320-9 32705365 PMC11804658

[43] Jin G., et al., DNA replication and sister chromatid cohesion 1 promotes breast carcinoma progression by modulating the Wnt/β-catenin signaling and p53 protein. Journal of Biosciences, 2020. 45(1): p. 1–11. 33184243

[44] Kim J.-T., et al., DNA Replication and Sister Chromatid Cohesion 1 (DSCC1) of the Replication Factor Complex CTF18-RFC is Critical for Colon Cancer Cell Growth. Journal of Cancer, 2019. 10(24): p. 6142–6153. doi: 10.7150/jca.32339 31762824 PMC6856584

[45] Sun Z., et al., PRAME is critical for breast cancer growth and metastasis abs. Gene, 2016. 594(1): p. 160–164. doi: 10.1016/j.gene.2016.09.016 27632898

[46] Al-Khadairi G., et al., PRAME promotes epithelial-to-mesenchymal transition in triple negative breast cancer. Journal of Translational Medicine, 2019. 17. doi: 10.1186/s12967-018-1757-3 30602372 PMC6317205

[47] Epping M.T., et al., PRAME expression and clinical outcome of breast cancer. British Journal of Cancer, 2008. 99(3): p. 398–403. doi: 10.1038/sj.bjc.6604494 18648365 PMC2527791

[48] Koppula P., et al., Amino acid transporter SLC7A11/xCT at the crossroads of regulating redox homeostasis and nutrient dependency of cancer. Cancer Communications, 2018. 38. doi: 10.1186/s40880-018-0288-x 29764521 PMC5993148

[49] Yang Y. and Yee D., IGF-I Regulates Redox Status in Breast Cancer Cells by Activating the Amino Acid Transport Molecule xC. Cancer Research, 2014. 74(8): p. 2295–2305. doi: 10.1158/0008-5472.CAN-13-1803 24686172 PMC4006361

[50] Koppula P., Zhuang L., and Gan B., Cystine transporter SLC7A11/xCT in cancer: ferroptosis, nutrient dependency, and cancer therapy. Protein & Cell, 2020. 10.1007/s13238-020-00789-5 PMC8310547 33000412

[51] Miguchi M., et al., Gasdermin C Is Upregulated by Inactivation of Transforming Growth Factor beta Receptor Type II in the Presence of Mutated Apc, Promoting Colorectal Cancer Proliferation. Plos One, 2016. 11(11). 10.1371/journal.pone.0166422 PMC5105946 27835699

[52] Hou J., et al., PD-L1-mediated gasdermin C expression switches apoptosis to pyroptosis in cancer cells and facilitates tumour necrosis. Nature Cell Biology, 2020. 22(10): p. 1264-+. doi: 10.1038/s41556-020-0575-z 32929201 PMC7653546

[53] Wei J., et al., Overexpression of GSDMC is a prognostic factor for predicting a poor outcome in lung adenocarcinoma. Molecular Medicine Reports, 2020. 21(1): p. 360–370. doi: 10.3892/mmr.2019.10837 31939622 PMC6896373

[54] Osthus R.C., et al., The Myc target gene JPO1/CDCA7 is frequently overexpressed in human tumors and has limited transforming activity in vivo. Cancer Research, 2005. 65(13): p. 5620–5627. doi: 10.1158/0008-5472.CAN-05-0536 15994934 PMC1224734

[55] Ye L., et al., Overexpression of CDCA7 predicts poor prognosis and induces EZH2-mediated progression of triple-negative breast cancer. International Journal of Cancer, 2018. 143(10): p. 2602–2613. doi: 10.1002/ijc.31766 30151890

[56] Andres S.A., et al., Interaction between smoking history and gene expression levels impacts survival of breast cancer patients. Breast Cancer Research and Treatment, 2015. 152(3): p. 545–556. doi: 10.1007/s10549-015-3507-z 26202054

[57] Chang Y.P., et al., Crystal structure of the GINS complex and functional insights into its role in DNA replication. Proceedings of the National Academy of Sciences of the United States of America, 2007. 104(31): p. 12685–12690. doi: 10.1073/pnas.0705558104 17652513 PMC1937527

[58] Rong Z., et al., GINS complex subunit 4, a prognostic biomarker and reversely mediated by Kruppel-like factor 4, promotes the growth of colorectal cancer. Cancer Science, 2020. 111(4): p. 1203–1217. doi: 10.1111/cas.14341 32012389 PMC7156840

[59] Yamane K., et al., Regulation of SLD5 gene expression by miR-370 during acute growth of cancer cells. Scientific Reports, 2016. 6. doi: 10.1038/srep30941 27499248 PMC4976388

[60] Camarda R., et al., Inhibition of fatty acid oxidation as a therapy for MYC-overexpressing triple-negative breast cancer. Nature Medicine, 2016. 22(4): p. 427-+. doi: 10.1038/nm.4055 26950360 PMC4892846

[61] Shahbandi A., Nguyen H.D., and Jackson J.G., TP53 Mutations and Outcomes in Breast Cancer: Reading beyond the Headlines. Trends in Cancer, 2020. 6(2): p. 98–110. doi: 10.1016/j.trecan.2020.01.007 32061310 PMC7931175

[62] Kim J.-Y., et al., The relationship between nuclear factor (NF)-kappa B family gene expression and prognosis in triple-negative breast cancer (TNBC) patients receiving adjuvant doxorubicin treatment. Scientific Reports, 2016. 6. doi: 10.1038/srep31804 27545642 PMC4992884

[63] Hao S., et al., New insights into the prognostic value of Ki-67 labeling index in patients with triple-negative breast cancer. Oncotarget, 2016. 7(17): p. 24824–24831. doi: 10.18632/oncotarget.8531 27050075 PMC5029745

[64] Munzone E., et al., Prognostic value of Ki-67 labeling index in patients with node-negative, triple-negative breast cancer. Breast Cancer Research and Treatment, 2012. 134(1): p. 277–282. doi: 10.1007/s10549-012-2040-6 22467243

[65] Asplund A., et al., Antibodies for profiling the human proteome-The Human Protein Atlas as a resource for cancer research. Proteomics, 2012. 12(13): p. 2067–2077. doi: 10.1002/pmic.201100504 22623277

[66] Paci P., et al., Integrated transcriptomic correlation network analysis identifies COPD molecular determinants. Scientific reports, 2020. 10(1): p. 3361–3361. doi: 10.1038/s41598-020-60228-7 32099002 PMC7042269

[67] Rich J.T., et al., A practical guide to understanding Kaplan-Meier curves. Otolaryngology-Head and Neck Surgery, 2010. 143(3): p. 331–336. doi: 10.1016/j.otohns.2010.05.007 20723767 PMC3932959

[68] Nagy A., et al., Validation of miRNA prognostic power in hepatocellular carcinoma using expression data of independent datasets. Scientific Reports, 2018. 8. 10.1038/s41598-018-27521-y PMC6003936 29907753

[69] Gyorffy B., Survival analysis across the entire transcriptome identifies biomarkers with the highest prognostic power in breast cancer. Computational and Structural Biotechnology Journal, 2021. 19: p. 4101–4109. doi: 10.1016/j.csbj.2021.07.014 34527184 PMC8339292

[70] Caldera M., et al., Interactome-based approaches to human disease. Current Opinion in Systems Biology, 2017. 3: p. 88–94.

[71] Khan A., et al., JASPAR 2018: update of the open-access database of transcription factor binding profiles and its web framework. Nucleic Acids Research, 2018. 46(D1): p. D260–D266. doi: 10.1093/nar/gkx1126 29140473 PMC5753243

[72] Mermel C.H., et al., GISTIC2.0 facilitates sensitive and confident localization of the targets of focal somatic copy-number alteration in human cancers. Genome Biology, 2011. 12(4). doi: 10.1186/gb-2011-12-4-r41 21527027 PMC3218867

[73] Aryee M.J., et al., Minfi: a flexible and comprehensive Bioconductor package for the analysis of Infinium DNA methylation microarrays. Bioinformatics, 2014. 30(10): p. 1363–1369. doi: 10.1093/bioinformatics/btu049 24478339 PMC4016708

