# Supplementary figures and images for: In silico recognition of a prognostic signature in basal-like breast cancer patients
Source: PLoS One. 2022 Feb 15;17(2):e0264024. doi: 10.1371/journal.pone.0264024 (PMC8846521; doi:10.1371/journal.pone.0264024)

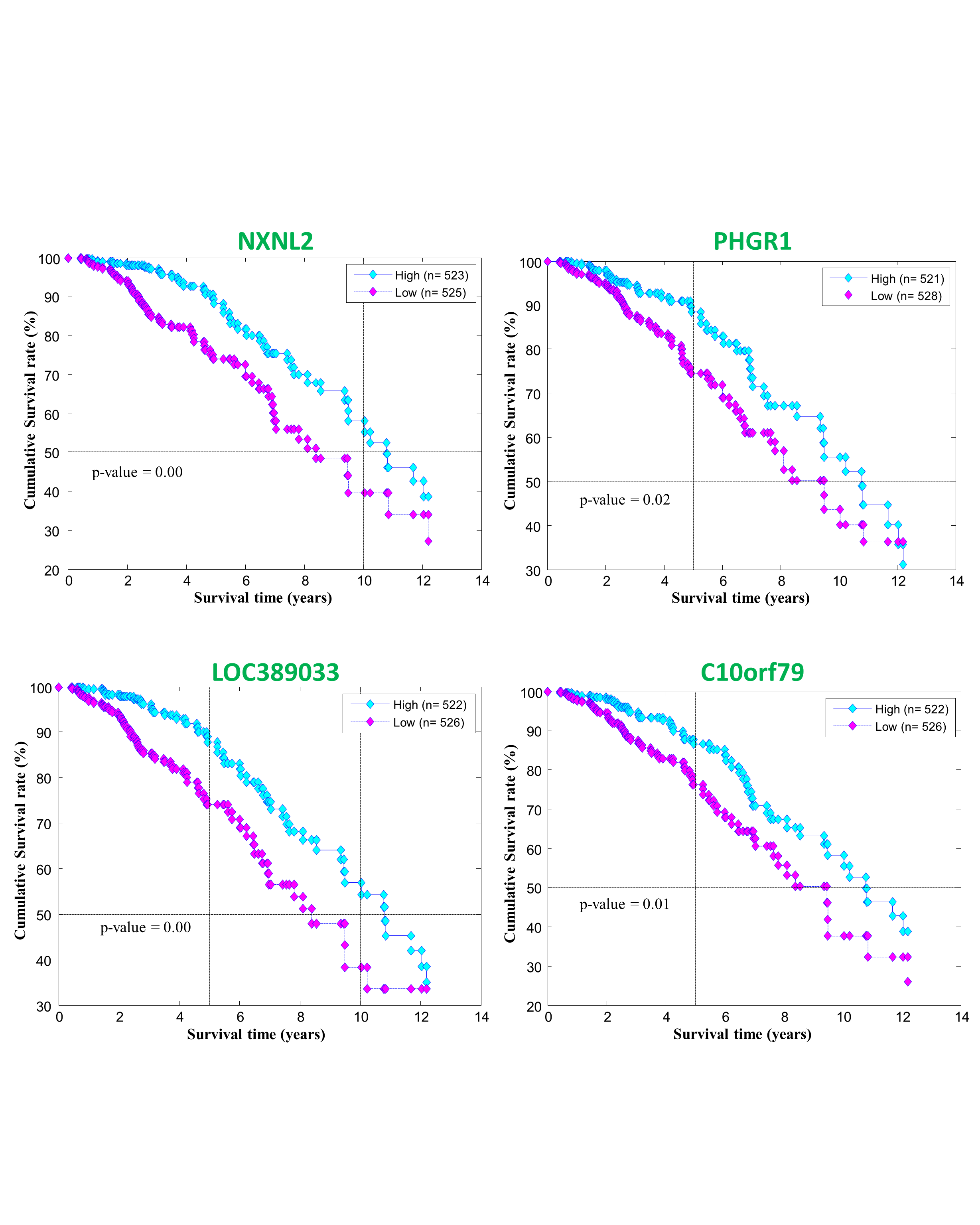

Supplement: S1 Fig — Kaplan-Meier analyzes to evaluate the correlations between the expression of the basal-like specific switch genes and the OS in TCGA breast invasive carcinoma patients. Low- and high-expression groups refer to patients with expression levels lower and greater than the 50th percentile, respectively. (PNG) [file pone.0264024.s001.png]

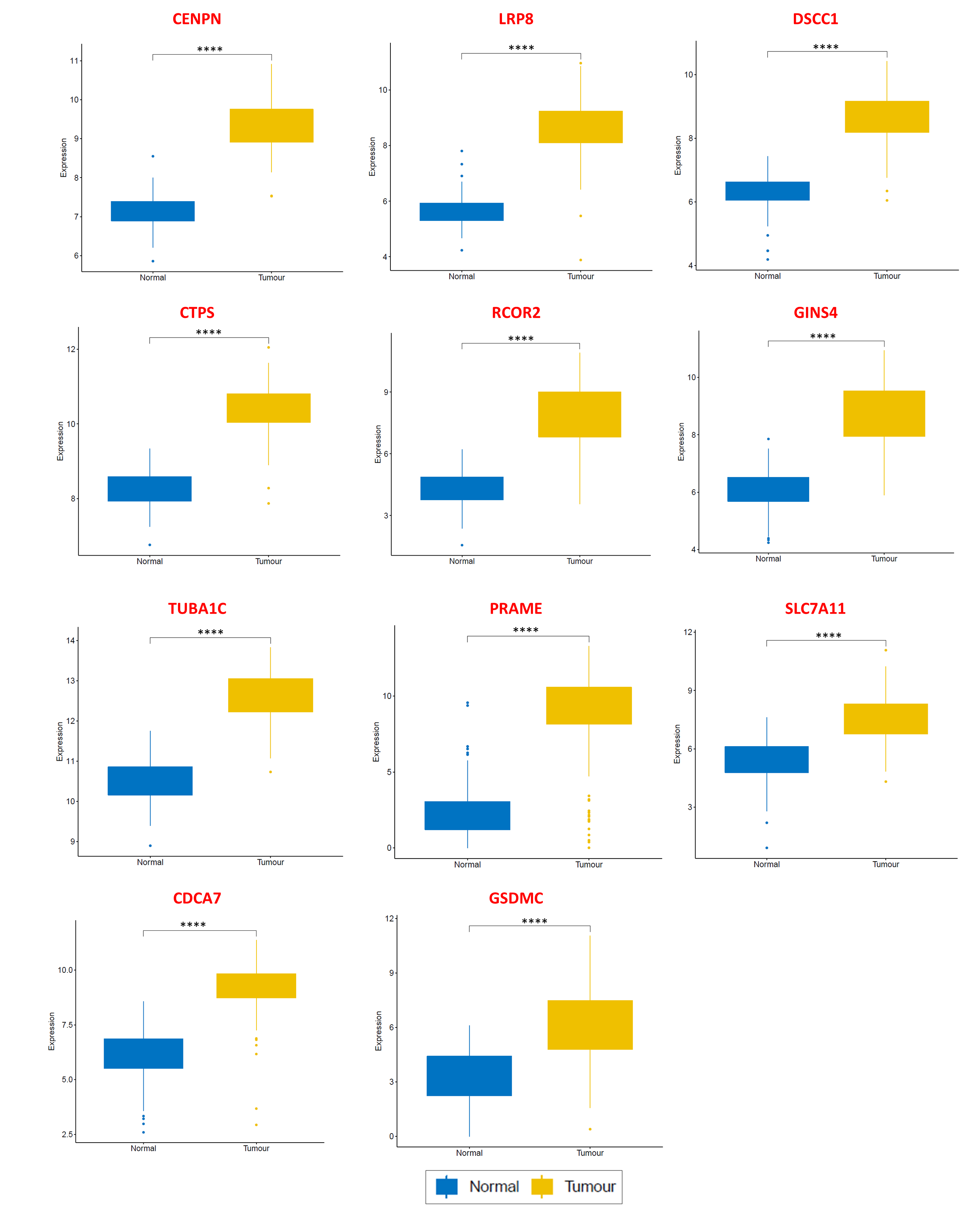

Supplement: S2 Fig — Gene expression levels of the 11 basal-like specific switch genes point out from the Kaplan-Meier survival analysis in basal-like and normal samples available from TCGA repository. T-test was used to compare the means of the selected genes between the two sample groups (Normal and Tumour) and statistical significance was indicated by the star symbols (i.e., ns: p > 0.05, *: p ≤ 0.05, **: p ≤ 0.01, ***: p ≤ 0.001, ****: p ≤ 0.0001). (PNG) [file pone.0264024.s002.png]
